# Acute-Subacute Paracoccidioidomycosis

**DOI:** 10.4269/ajtmh.24-0563

**Published:** 2025-03-04

**Authors:** Carlos McFarlane, Omayra Chincha, Carlos Seas

**Affiliations:** ^1^ Facultad de Medicina Alberto Hurtado, Universidad Peruana Cayetano Heredia, Lima, Peru;; ^2^ Departamento de Enfermedades Infecciosas, Tropicales, y Dermatologicas, Hospital Nacional Cayetano Heredia, Lima, Peru;; ^3^ Instituto de Medicina Tropical “Alexander von Humboldt,” Universidad Peruana Cayetano Heredia, Lima, Peru

**Figure 1.**
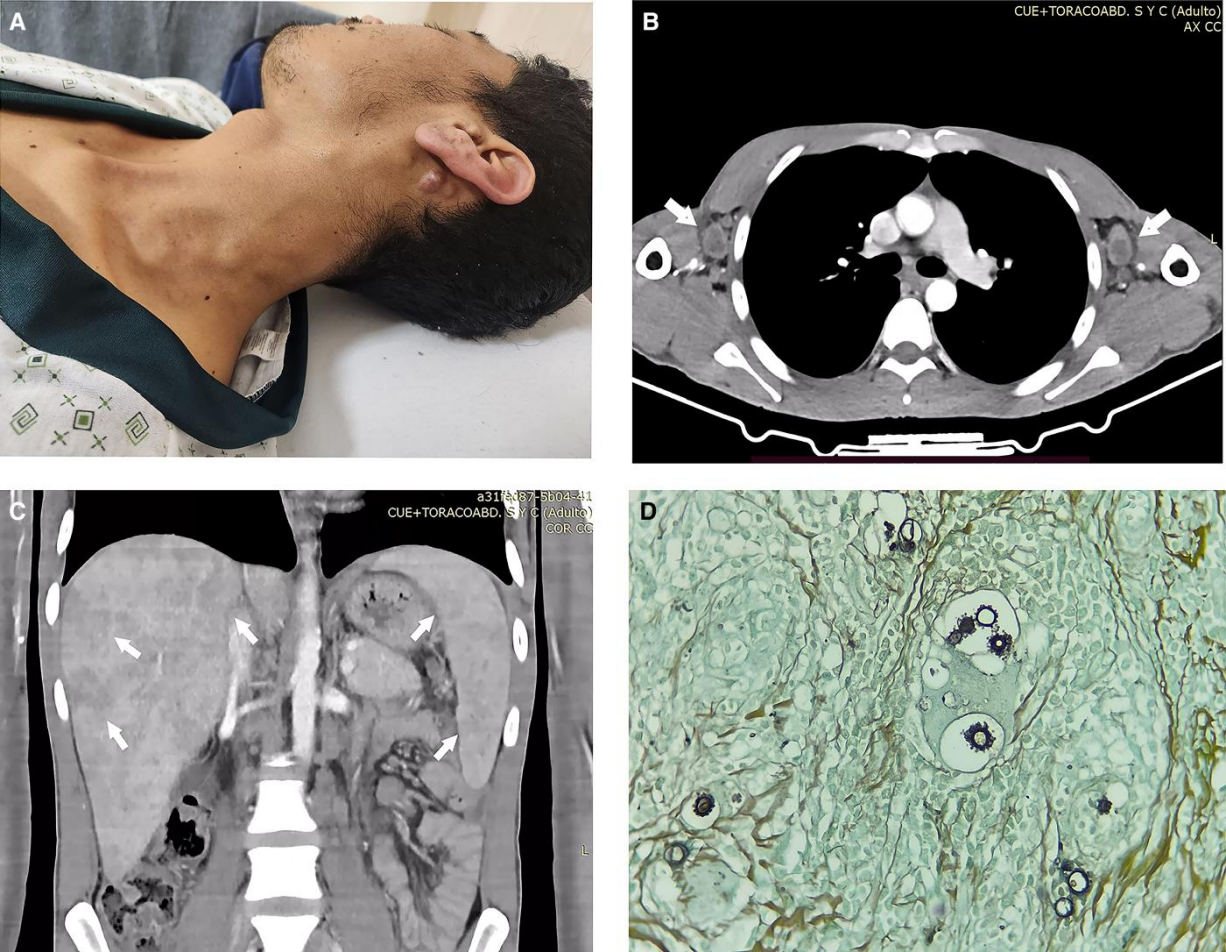
(**A**) Enlarged cervical lymph nodes, (**B**) chest computed tomography (CT) showing bilateral axillary lymph node enlargement (arrows), (**C**) abdominal CT showing liver and spleen abscesses (arrows), (**D**) histopathological examination of cervical lymph nodes stained with Grocott methenamine silver showing multiple budding yeast compatible with *Paracoccidioides* species.

A 29-year-old male farmer from Yambrasbamba, Amazonas, in the Amazon rainforest in Peru presented with a 4-month history of cervical lymphadenopathy, persistent fever, jaundice, weight loss, abdominal pain, and malaise. Physical examination revealed multiple mobile and painless lymph nodes in the submental, submandibular, and cervical chains (Figure 1A). Jaundice and hepatosplenomegaly were present. Laboratory tests showed microcytic hypochromic anemia (hemoglobin: 9 g/dL), leukocytosis (15,600 cells/mm^3^) with lymphocytosis (neutrophils 35.8%, eosinophils 0.5%, basophils 0.8%, monocytes 2.2%, and lymphocytes 60.7%), elevated alkaline phosphatase (1,177 U/L), and elevated total bilirubin (14 mg/dL; direct bilirubin 12.6 mg/dL). Serologies for HIV, human T-lymphotropic virus-1, syphilis, hepatitis C, and hepatitis B were negative. A chest computed tomography (CT) scan revealed cervical and axillary lymphadenopathy without pulmonary involvement (Figure 1B). Abdominal CT showed hepatosplenomegaly with multiple liver and spleen abscesses and enlargement of mesenteric lymph nodes (Figure 1C). Histopathology of a cervical lymph node biopsy demonstrated chronic granulomatous inflammation with abundant giant cells. Periodic acid–Schiff and Grocott methenamine silver stains confirmed the presence of fungal structures consistent with *Paracoccidioides* species (Figure 1D). The patient was initially treated with amphotericin B deoxycholate for 2 weeks, followed by trimethoprim-sulfamethoxazole at discharge. By that time, the fever, abdominal pain, and malaise had resolved. Partial improvement in jaundice was observed, with a reduction in alkaline phosphatase levels (821 U/L) and total bilirubin (3.3 mg/dL). This presentation corresponds to the acute-subacute form (AF) of paracoccidioidomycosis, also known as the juvenile form, characterized by rapid onset of symptoms. Clinical manifestations are related to the involvement of the mononuclear phagocytic system, with lymphadenopathies in superficial and/or deep lymph nodes being the most common presentation. Although typically associated with younger individuals, the AF can also occur in adults and is usually severe because of the rapid progression of the disease and significant involvement of the mononuclear system, leading to a marked depression of cell-mediated immune response.^1^^,^^2^
